# Fungal β-1,3-glucan synthase: a review of structure, mechanism, and regulation

**DOI:** 10.1093/femsyr/foaf071

**Published:** 2025-11-28

**Authors:** Xintong Huang, Muwu Chen, Zan Chen, Yueping Zhang

**Affiliations:** State Key Laboratory of Veterinary Public Health and Safety, College of Veterinary Medicine, China Agricultural University, Beijing 100193, China; College of Biological Sciences, China Agricultural University, Beijing 100193, China; State Key Laboratory of Veterinary Public Health and Safety, College of Veterinary Medicine, China Agricultural University, Beijing 100193, China; State Key Laboratory of Veterinary Public Health and Safety, College of Veterinary Medicine, China Agricultural University, Beijing 100193, China

**Keywords:** fungal cell wall, antifungal drug resistance, β-1,3-glucan synthase, Fks, Cryo-EM

## Abstract

Fungal β-1,3-glucan synthase (Fks) plays a central role in synthesizing β-1,3-glucan, the main structural polysaccharide of fungal cell walls, and serves as a key target for antifungal drugs, such as echinocandins and ibrexafungerp. Recent cryo-electron microscopy (cryo-EM) studies have revealed the architecture of the Fks1 and Fks1–Rho1 complex and provided new insights into its catalytic and regulatory mechanisms. This review summarizes current understanding of Fks, including its domain organization, transmembrane topology, conformational dynamics, and evolutionary comparison with structurally resolved glycosyltransferases (GTs), including bacterial cellulose synthase (BcsA), plant cellulose synthase (CesA), and other eukaryotic GTs. Through comparison of publicly available cryo-EM structures of Fks in both the apo-state and Rho1-bound state, a working mechanism of the activated Fks has been discussed. In addition, we present a potential gating model of β-glucan translocation and drug-inhibition by integrating literature with structure-based analyses. This review provides a structure-based functional model of fungal β-1,3-glucan synthase and the putative binding mechanism of its inhibitor, aiming to support future antifungal drug discovery.

## Teaser

This review combines cryo-EM structures and molecular modeling to reveal how fungal enzymes build cell walls, resist antifungal drugs like echinocandins by altering specific binding sites, and diverge evolutionarily, guiding future therapies against resistant infections.

## Introduction

Invasive fungal infections pose a significant global health burden, driving the urgent need for novel antifungal agents. The fungal cell wall, absent in humans, represents an ideal target for selective therapy. β-1,3-glucan is the major polysaccharide and primary structural component of the cell wall in many fungi, including important model organisms like *Saccharomyces cerevisiae* and human pathogens such as *Candida albicans, Aspergillus fumigatus*, and others. It forms the core fibrillar network of the cell wall, providing rigidity and structural strength (Lesage and Bussey 2006, Gow and Lenardon 2023).

Synthesis of this essential polymer is catalysed by fungal β-1,3-glucan synthase (GS or Fks protein), a key enzyme complex localized to the plasma membrane. Its core function is to utilize uridine diphosphate glucose (UDP-Glc) as a glycosyl donor to catalyze the synthesis of linear β-1,3-glucan polymers, a process involving the formation of β(1→3) glycosidic bonds. The synthesized glucan chains are subsequently extruded across the plasma membrane into the periplasmic space (Douglas et al. 1994). In the periplasm, these linear β-1,3-glucan chains undergo further modifications, such as elongation, branching, and cross-linking with other cell wall components (e.g. β-1,6-glucan, chitin, and mannoproteins), ultimately integrating into the complex cell wall structure (Wang et al. 2018).

Targeting Fks is a validated strategy for antifungal drug development. Genetic studies unequivocally demonstrate the essentiality of Fks for fungal viability: simultaneous deletion of *FKS1* and *FKS2* is lethal in *S. cerevisiae* (Ha et al. 2006), and the major *FKS* genes are essential in pathogenic fungi like *C. albicans, Cryptococcus neoformans* (Thompson et al. 1999) and *Coccidioides posadasii* (Kellner et al. 2005). Consequently, Fks is the specific molecular target for several important classes of antifungal drugs currently in clinical use, including echinocandins (e.g. caspofungin, micafungin, and anidulafungin) and the newer triterpenoid drug ibrexafungerp (Denning, 2003).

However, the rise of antifungal resistance, particularly multidrug-resistant strains like *Candida auris*, underscores the limitations of current therapies and the critical need for deeper mechanistic insights into Fks. Understanding the molecular basis of Fks function, inhibition, and resistance emergence is paramount for developing next-generation antifungals.

## Molecular component of the β-1,3-glucan synthase complex

### Subunit composition

Functional β-1,3-glucan synthase is generally considered a multisubunit complex. Its core composition includes at least a catalytic subunit Fks and a regulatory subunit Rho1 (Li et al. 2025). Rho1 is a small GTPase belonging to the Ras superfamily, loosely associated with the cell membrane (Humphries et al. 2020). Only when Rho1 is in its GTP (Guanosine Triphosphate)-bound activated state can it activate the catalytic subunit Fks (Chhetri et al. 2020, Hu et al. 2023). Prenylation of Rho1 is necessary for its membrane localization and function in activating glucan synthase (Cappellaro et al. 1998).

Early biochemical separation experiments also support this multicomponent model. Studies found that soluble fractions (potentially containing Rho1) or membrane fractions (containing Fks) alone exhibit low or no activity, and enzyme activity can only be restored upon reconstitution of both fractions (Douglas 2001). GTP analogs protect the activity of the soluble fraction, while UDP (Uridine Triphosphate)-glucose protected the membrane fraction (Douglas 2001). Additionally, evidence suggests that other unknown components may be involved in the complex. However, the exact subunit stoichiometry and oligomeric state of the enzyme complex remain unclear (Douglas 2001, Chhetri et al. 2020).

### Catalytic subunit FKS

The catalytic subunit Fks is encoded by the *FKS* gene family (also previously named *GSC, ETG, CND, CWH, PBR, GLS*, and so on). The copy number of *FKS* genes varies among different fungal species. *Saccharomyces cerevisiae* possesses three homologous genes: *FKS1, FKS2*, and *FKS3* (Wang et al. 2018). *FKS1* encodes the enzyme primarily active during vegetative growth. *FKS2* expression becomes important under stress conditions (e.g. high temperature and glucose limitation) or during sporulation. *FKS3* is mainly involved in spore wall formation and may affect Rho1 stability but contributes little to *in vitro* GS activity (Zajac et al. 2025). The proteins encoded by *FKS1* and *FKS2* share about 88% homology and exhibit partial functional redundancy, as the double knockout is lethal (Mazur et al. 1995). *Candida albicans* also has three homologous genes (*FKS1/GSC1, FKS2/GSL1*, and *FKS3/GSL2*) (Garcia-Rubio et al. 2020). Among these, *FKS1* is essential and encodes the major catalytic subunit (Thompson et al. 1999). Although *FKS2* and *FKS3* are expressed, they appear to primarily function as negative regulators of *FKS1* and influence cellular sensitivity to echinocandins (Suwunnakorn et al. 2018). Filamentous fungi (e.g. *Aspergillus* spp. and *C. posadasii*) and *C. neoformans* typically contain only a single *FKS* gene. This single *FKS* gene is essential for viability (Liu and Balasubramanian, 2001).

This variation in *FKS* gene copy number reflects different evolutionary strategies. Gene duplication allows for functional specialization or subfunctionalization. In *S. cerevisiae*, Fks1 handles vegetative growth, Fks2 manages stress and sporulation, and Fks3 participates in spore wall construction, indicating adaptation to different demands for glucan synthesis during various life cycle stages or environmental conditions (Ishihara et al. 2007). In *C. albicans*, the essential Fks1 and the negatively regulating Fks2/Fks3 (Suwunnakorn et al. 2018) form a more complex regulatory network, possibly for fine-tuning glucan synthesis or drug response. In contrast, filamentous fungi rely on a single Fks enzyme, implying that the regulation of this single enzyme’s activity, localization, and so on, might need to be more intricate, or that these fungi have relatively smaller variations in glucan structure or synthesis rate requirements across different growth stages. The essentiality of the single *FKS* gene theoretically makes these species more sensitive to Fks inhibitors (Liu and Balasubramanian, 2001), although other factors (like cell wall composition differences) also affect actual drug sensitivity (Thompson et al. 1999). This difference in gene copy number may influence drug efficacy and the evolution of resistance mechanisms in different fungal groups.

Fks proteins are generally large, with molecular weights ranging from 180 to 280 kDa (Yang et al. 2023) (Table 1). For example, *S. cerevisiae* Fks1 contains 1876 amino acid residues (Hu et al. 2023), while *C. posadasii* Fks1 is predicted to contain 1902 amino acids with a molecular weight of ~217 kDa (Kellner et al. 2005). Fks proteins are integral membrane proteins with multiple transmembrane helices (TMHs). Recent cryo-electron microscopy (cryo-EM) structure determination of *S. cerevisiae* Fks1 revealed that it actually contains 17 TMHs (Hu et al. 2023). These TMHs are often described as clustered into two regions, separated by a large hydrophilic loop/domain facing the cytoplasm.

**Table 1.** Summary of cryo-EM structures of β-1,3-glucan synthase.

Species	Protein state	Resolution (Å)	PDB ID	Key findings	References
*S. cerevisiae* Fks1	Apo	3.4	7XE4	Active site at interface, translocation channel, and lipid interactions	Hu et al. (2023)
*S. cerevisiae* Fks1	S643P mutant (+UDP-Glc, Caspo)	3.5	7YUY	Resistance hotspot localization, altered lipid arrangement near mutation site, and elongated density in channel	Hu et al. (2023)
*S. cerevisiae* Fks1	Apo	2.47	8JZN	Cellulose synthase-like fold, GT-A domain, 15 TMs, closed channel, key disulfide bonds, GT-A fold, 15 TMs, DAN motif, metal-ion independent, and closed channel	Zhao et al. ( 2023)
*S. cerevisiae* Fks1	Apo (resting state)	3.16	8WL6	Cellulase-like conformation, comparison with Rho1-bound state	Li et al. (2025)
*S. cerevisiae* Fks1	Fks1–Rho1 complex (activated state)	3.40	8WLA	Rho1 binding site, Rho1-induced conformational changes, proposed activation mechanism involving “finger helix” pushing glucan	Li et al. (2025)

### Regulatory subunit Rho1

**Figure 1.**
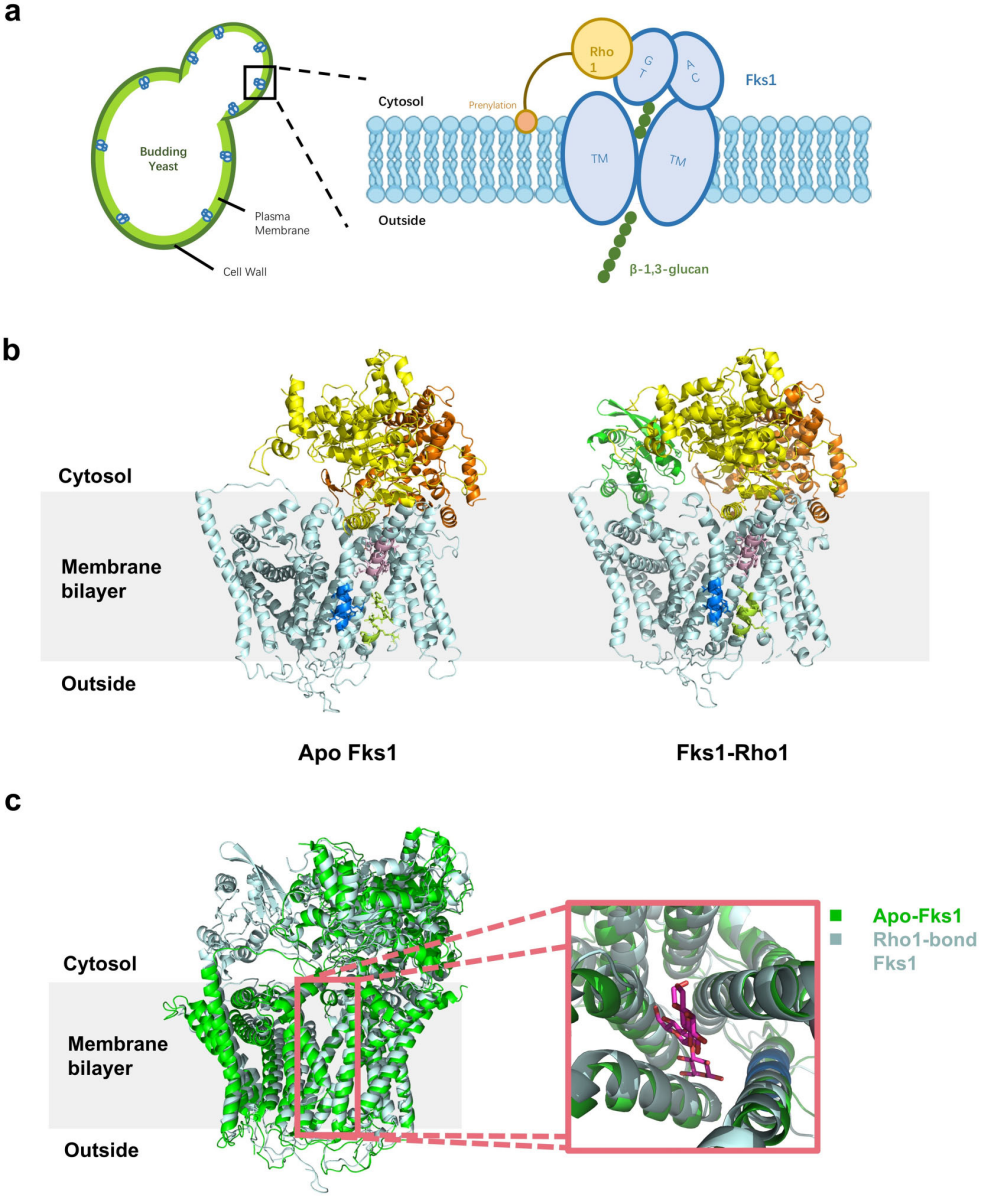
The structure and membrane localization of fungal β-1,3-glucan synthase in *S. cerevisiae*. (a) Left: schematic of a budding yeast cell, showing the plasma membrane (light green layer) and cell wall (green layer). Blue dots on the membrane indicate the membrane-embedded β-1,3-glucan synthase complex. Right: zoom-in of a single glucan synthase complex. The catalytic subunit Fks1 spans the plasma membrane with multiple transmembrane helices (TM). Fks1 contains a cytosolic glycosyltransferase domain (GT) and an accessory domain (AC) for potential protein interactions or regulation. The GTPase Rho1 (yellow) regulates Fks1 activity through its GTP-bound form and requires prenylation (orange circle) for membrane localization. The enzyme synthesizes and extrudes linear β-1,3-glucan (green dots) across the membrane. (b) The apo-Fks1 structure (PDB:8WL6) and Rho1–Fks1 complex structure (PDB:8WLA). (c) Superposition of apo-Fks1 and Rho1-bound Fks1 structures. Compared with the apo state, the Rho1-bound conformation exhibits a markedly narrower glucan-translocating channel. The electron density map from cryo-EM shows the glucan chain (purple) positioned within the glucan-translocating channel (PDB: 7YUY).

Rho1, a small GTPase of the Ras superfamily, serves as the critical regulatory subunit of GS. It cycles between an active GTP-bound state and an inactive GDP-bound state, with only the GTP-bound form capable of activating the catalytic subunit Fks to drive glucan synthesis (Li et al. 2025). Rho1 requires posttranslational prenylation (typically farnesylation or geranylgeranylation) for proper membrane localization, enabling its interaction with Fks at the plasma membrane (Arellano et al. 1996) (Fig. 1a).

Structurally, Rho1 contains conserved GTPase domains responsible for nucleotide binding/hydrolysis and effector interaction, along with a hypervariable C-terminal region that mediates membrane anchoring and protein–protein interactions (Li et al. 2025). Beyond its role in GS activation, Rho1 integrates signals from the Cell Wall Integrity (CWI) pathway, coordinating glucan synthesis with stress responses (e.g. osmotic or oxidative stress) and fungal morphogenesis. In pathogenic fungi, Rho1 is essential for virulence, as it ensures robust cell wall construction to evade host immune recognition. Its evolutionary divergence from human Rho homologs (e.g. RhoA) and functional indispensability (Müller et al. 2020) make Rho1 a promising antifungal target, particularly for compounds disrupting its GTPase activity or interaction with Fks. Through structural comparison, binding of Rho1 appears to induce a potential constriction of the glucan translocation channel, akin to a peristaltic-like gating mechanism that facilitates glucan chain elongation and extrusion (Fig. 1c).

In summary, β-1,3-glucan synthase is a core complex composed of the catalytic subunit Fks and the regulatory subunit Rho1. Fks is responsible for catalyzing glucan synthesis, and its gene copy number varies among fungi. Rho1 acts as a GTPase switch, regulating Fks activity via its GTP-bound state, and its membrane localization depends on prenylation.

## High-resolution structure determination of Fks protein

### Cryo-EM structure of *S. cerevisiae* Fks1

Recent breakthroughs in resolving the structures of large membrane protein complexes have been achieved using cryo-EM. Several research groups have successfully determined the high-resolution structure of *S. cerevisiae* Fks1, including its apo state (unbound to substrate or regulators) and certain mutant forms. Reported resolutions have reached levels of 2.47 Å (Zhao et al. 2023), 3.4 Å (Hu et al. 2023), and 3.5 Å (Hu et al. 2023). These structures represent the first high-resolution structures of members of the Glycosyltransferase 48 (GT48) family, providing an unprecedented structural basis for understanding their functional mechanisms (Hu et al. 2023, Li et al. 2025, Zhao et al. 2023).

### Overall fold and domain organization

The structure of *S. cerevisiae* Fks1 measures ~105 Å in height and 95 Å in width, and can be broadly divided into a transmembrane (TM) region and a large cytosolic region (Hu et al. 2023, Li et al. 2025,Zhao et al. 2023). Its core catalytic region exhibits a fold similar to bacterial cellulose synthase BcsA and plant cellulose synthase CesA (belonging to the GT-2 family), known as the “cellulose synthase-like fold” (Hu et al. 2023). This reveals an evolutionary link between Fks (GT48 family) and GT-2 family enzymes in their core structure.

Fks1’s cytosolic region contains an N-terminal Accessory Domain (AC domain, approximate residues 146–442) and a large GT domain (approximate residues 713–1281/1294), which is inserted between TM6 and TM7. The GT domain displays a typical Glycosyltransferase-A fold (GT-A) type α/β/α sandwich fold. The TM region contains 17 TM helices (TM1–17). These TM helices are roughly divided into two groups (TM1–6 and TM7–17). The TM7–17 region is tilted relative to the TM1–6 region, forming a large cavity facing the cytosol in the middle of the structure. This region also contains two amphipathic horizontal helices (HH1 and HH2) and several interface helices (IF1, IF2, and IF3) (Hu et al. 2023, Li et al. 2025, Zhao et al. 2023).

### Catalytic site

The catalytic active site of Fks1 is located within the cytosolic GT domain, near the membrane–cytosol interface (Hu et al. 2023, Li et al. 2025, Zhao et al. 2023). It forms a large horizontal groove or solvent-exposed chamber. Through structural comparisons with other GTs like BcsA and site-directed mutagenesis experiments, researchers have identified several key catalytic and substrate-binding residues. These include the catalytically essential ED motif (E1221 and D1222 in *S. cerevisiae* Fks1) and residues involved in UDP-Glc binding (e.g. Y849, E851, K1082, and N1085). D1222 corresponds to the third aspartate in the conserved D,D,D signature motif of cellulose synthases. An interesting finding is that Fks1 possesses a Asparagine-Aspartic acid-Asparagine motif (DAN) motif (D1102-A1103-N1104) at the position corresponding to the conserved DxD motif found in many metal-ion-dependent GT-A enzymes (Zhao et al. 2023). D1102 corresponds to the second aspartate of the cellulose synthase D,D,D signature. Mutational studies confirmed that this DAN motif is crucial for Fks1 function. Consistent with this structural feature, biochemical experiments indicate that Fks1 activity is independent of metal ions (like Mg^2+^) (Zhao et al. 2023).

### Glucan translocation channel

Cryo-EM structures revealed a putative TM glucan translocation channel located directly beneath the catalytic active site, spanning the entire membrane bilayer. This channel is primarily formed by TM5–TM10 or TM5–8, TM11–12. In the resolved apo state structures, this channel is closed on the extracellular side. Compared to bacterial cellulose synthase BcsA, TM8 of Fks1 is shifted inward, occupying the glucan transport path and leading to a closed channel in the apo state. The proposed translocation mechanism involves conformational changes triggered by substrate binding. These changes might include the outward movement of TM8 and the downward movement of the GT domain, thereby opening the channel and extruding the nascent glucan chain out of the cell. The relatively flexible interface helices IF2/IF3 and a gating loop in the apo structure might play roles in stabilizing the growing glucan chain and controlling channel entry (Hu et al. 2023, Zhao et al. 2023). The structure also shows hydrophilic pockets on both the cytosolic and extracellular sides of the channel region; their juxtaposition leads to significant local thinning of the membrane, potentially lowering the energy barrier for TM glucan translocation (Zhao et al. 2023). In the structure of the fks1-S643P mutant (resolved in the presence of substrate and inhibitor), elongated electron density was observed within the channel, further supporting its function as a product channel (Hu et al. 2023) (Fig. 1c).

### Other structural features

The structure shows two conserved disulfide bonds in the extracellular region of Fks1: one in the extracellular loop EL3 between TM5 and TM6 (connecting Cys658 and Cys669), and another in the extracellular loop EL4 between TM7 and TM8 (connecting Cys1328 and Cys1345) (Zhao et al. 2023). These disulfide bonds are proven essential for enzyme activity, possibly by restricting the flexibility of the extracellular loops. Additionally, the structure demonstrates interactions between Fks1 and membrane components, evidenced by bound lipid molecules (sterols and phospholipids) and pronounced membrane deformation (Hu et al. 2023, Zhao et al. 2023). These lipids are predominantly localized around the TM core formed by TM5–13 helices, suggesting a stabilizing role in the tilted orientation of these helices (Hu et al. 2023). The Rho1 Interaction site, as determined by structural studies of the Fks1–Rho1 complex, occupies a pocket formed by the GT domain and the TM7–15 helix cluster. Key interaction motifs include the R862-V870 sequence in the GT domain and the TM15-HH1 loop within the C-terminal domain (Zhao et al. 2023). Rho1 binding triggers substantial conformational rearrangements in Fks1, as supported by functional studies (Li et al. 2025).

### Structure of Fks1–Rho1 complex

Rho1, a small GTPase, regulates the catalytic activity of Fks1 through GTP-dependent conformational changes. Recent cryo-EM studies have resolved the high-resolution structure (3.40 Å) of the *S. cerevisiae* Rho1–Fks1 complex, elucidating their molecular binding basis and activation mechanisms (Li et al. 2025).

Rho1 binds as a monomer to a cytoplasmic cleft of Fks1, positioned between the GT domain and four lateral helices (TM7–TM15), forming a tight interface1. The GTP-binding domain of Rho1 (α3 helix region) interacts with Fks1’s RIL loop (residues Q1516, H1518, and so on) via hydrophilic interactions. Key residues include E98, E102, and Q113 in Rho1. Mutations at these sites significantly destabilize the complex and impair enzymatic activity (Hu et al. 2023, Li et al. 2025).

GTP binding induces conformational changes in Rho1, enhancing its affinity for Fks1. For instance, the constitutively active Rho1-Q68H mutant activates Fks1 even in the absence of GTPγS, indicating that GTP hydrolysis is critical for regulation. Crosslinking experiments (BS3) confirmed direct Rho1–Fks1 interaction, with GTPγS promoting complex formation (Li et al. 2025).

Structural comparison between apo-Fks1 and Rho1-bound Fks1 revealed significant displacements in the GT domain and TM helices (TM7–TM15). These changes likely facilitate glucan chain elongation and secretion through the membrane channel. A “ratchet and pawl” model was proposed: Rho1 drives stepwise conformational adjustments in Fks1’s GT domain via cyclic GTP/GDP binding, akin to a mechanical ratchet, ensuring continuous β-1,3-glucan synthesis (Li et al. 2025).

The Rho1–Fks1 complex structure unveils the core regulatory mechanism of fungal cell wall synthesis, providing critical residue and dynamic conformation insights for antifungal drug design. Targeting the α3 helix of Rho1 or the RIL loop of Fks1 with small-molecule inhibitors could disrupt their interaction and block glucan production.

**Figure 2.**
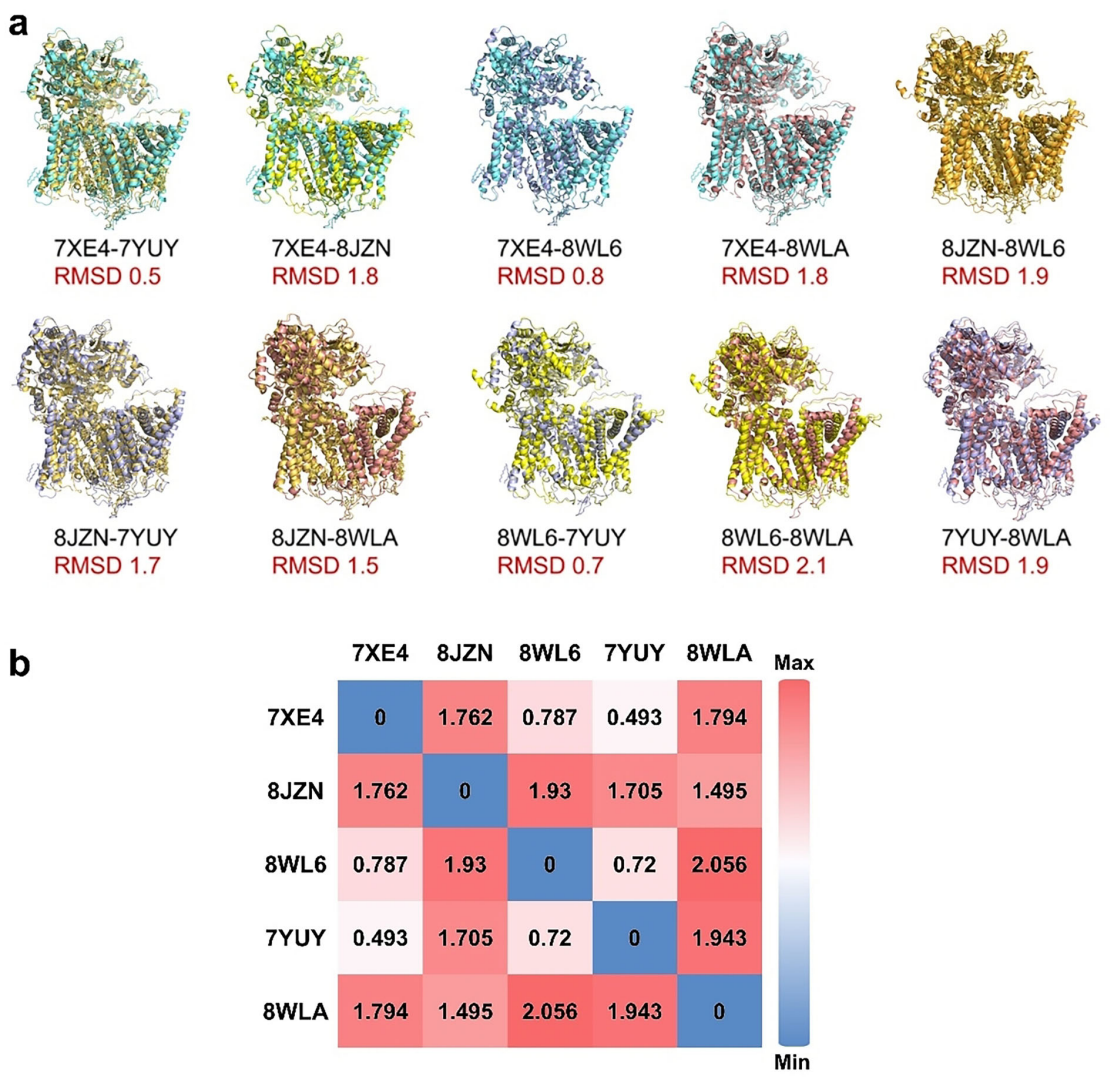
Structural comparison of cryo-EM-derived *S. cerevisiae* Fks1 monomers highlights conformational flexibility. (a) Structural superposition of five Fks1 monomers, including three wild-type structures (7XE4, 8JZN, and 8WL6), the S643P mutant (7YUY), and the Fks1 protomer extracted from the Rho1–Fks1 complex, is shown via pairwise alignments; each RMSD value quantifies the conformational deviation between the compared structures. (b) A heatmap of RMSD values for all structure pairs summarizes the degree of structural variability. Warmer colors indicate larger deviations.

Root-mean-square deviation (RMSD) analysis across the available *S. cerevisiae* Fks1 structures shows that the core fold is highly conserved (overall RMSD <2.0 Å) (Fig. 2). The structure of the activated *S. cerevisiae* Fks1–Rho1 complex (8WLA) shows the largest deviation, reflecting the significant conformational changes upon activation. Minor variations observed between different resting-state structures likely reflect the influence of different detergents used during purification, highlighting the inherent flexibility of the TM helices within the lipid microenvironment (Hu et al. 2023, Li et al. 2025).

Based on these structural features, a dynamic “molecular gearbox” model has been proposed (Li et al. 2025). Fks1 functions not as a static channel but as a machine, where coordinated conformational changes drive active transport of the elongating glucan chain. The enzyme synthesizes the polymer on the cytosolic membrane side and transports it to the periplasm through mechanochemical coupling, akin to interlocking gears converting catalytic energy into mechanical work. The proximity of the catalytic site to the membrane directly links chemical reactions to the initiation of gear-like movements, with the TM channel serving as both transport pathway and dynamic housing for the gear system. The closed channel in the apo state suggests a safety-locking mechanism to prevent substrate leakage. Observed domain movements reveal that the GT domain and TM helices (e.g. TM8) act as meshed transmission gears, whose rotational angles are precisely regulated by substrate binding and catalysis. Notably, membrane thinning through lipid interactions may optimize gear operation efficiency by reducing frictional resistance. This “gear assembly” model posits that glucan chain synthesis and transport are achieved via mechanochemical coupling: substrate binding triggers motion in the primary drive gear (GT domain), which propagates mechanical force through the TM helix gear train, ultimately opening the channel to complete TM delivery. This model can be contextualized alongside other processive enzymes like bacterial cellulose synthase (BcsA), which also utilizes a ratchet-like mechanism driven by a regulatory domain to couple synthesis with translocation (Nunnally et al. 2019). Despite shared mechanochemical coupling principles, Fks1 exhibits unique adaptations, notably in its dependence on the eukaryotic GTPase Rho1 (as opposed to the bacterial second messenger c-di-GMP) for regulatory input and in the composition of its “gear” components. High-resolution structures of Fks1 reveal a complex fold with TM and cytosolic regions (containing GT and AC domains), featuring a “cellulose synthase-like” core. Structural analysis identified the catalytic site, a metal-ion-independent DAN motif, a putative glucan translocation channel, and the interaction site for the regulatory subunit Rho1. Structural comparisons indicate Fks1 exists in resting and activated conformations, with dynamic changes potentially driving glucan synthesis and TM transport via a “gear-like” mechanism.

## Catalytic mechanism of β-1,3-glucan synthesis

### Substrate recognition and binding

β-1,3-Glucan synthase utilizes UDP-Glc as the glycosyl donor substrate. The catalytic active site, located at the cytosol–membrane interface (see details in Catalytic Site), is responsible for binding UDP-Glc. Structural studies have identified potential key residues involved in binding (e.g. Y849, E851, K1082, and N1085 in *S. cerevisiae* Fks1; Hu et al. 2023). Additionally, a conserved RXTG motif (RITG in *C. posadasii* Fks1, amino acids 1569–1572) is also considered part of the UDP-glucose binding site (Kellner et al. 2005). Kinetic analysis of partially purified *S. cerevisiae* GS showed a Michaelis constant (Km) for UDP-Glc of ~0.37 ± 0.11 mM, which is consistent with the intracellular UDP-Glc concentration range in yeast (0.15–0.3 mM) (Chhetri et al. 2020).

### Initiation of the glucan chain

Whether the Fks enzyme performs *de novo* synthesis initiation or requires a primer or acceptor molecule to start chain synthesis has long been an unresolved question. The enzyme’s processive nature, i.e. its ability to significantly extend a chain before dissociation, is generally accepted (Douglas 2001).

Although the provided materials lack direct experimental evidence definitively resolving the *de novo* versus primer initiation debate, recent structural and kinetic data show the enzyme’s high processivity, capable of synthesizing very long chains (see details in Elongation of the Glucan Chain and Determination of Product Length). This might favor a model where initiation and elongation are tightly coupled, possibly involving specific structural features or protein interactions not yet fully elucidated. The term “homopolymerization” has been used to describe this process (Chhetri et al. 2020), which could imply *de novo* initiation but is not conclusive proof.

### Elongation of the glucan chain

Fks enzyme catalyses the continuous addition of glucose units from UDP-Glc onto the growing β-1,3-glucan chain (Douglas 2001). Recent studies using substrate analogs and size-exclusion chromatography coupled with pulsed amperometric detection and radiation counting (SEC–PAD–RC) have clearly demonstrated that chain elongation occurs at the nonreducing end of the polymer chain (Chhetri et al. 2020).

Presteady-state kinetic analysis of purified *S. cerevisiae* GS allowed the first direct measurement of its chain elongation rate, approximately 50–51.5 s^−1^. This is the first reported chain elongation rate for a nucleotide sugar-dependent polysaccharide synthase (Chhetri et al. 2020). This rate is remarkably fast, over 100 times faster than the reported rates for β-glucanosyltransferases involved in cell wall remodeling (Johnson et al. 2011). This suggests that GS itself might be the primary determinant of the final polymer length.

### Determination of product length

The understanding of the length of β-1,3-glucan products synthesized by Fks enzyme has undergone significant revision.Early studies based on crude extract analysis suggested that the synthesized glucans were relatively short, yielding products with a degree of polymerization (DP) of approximately 60–80 glucose units. (Shematek et al. 1980). This led to early models proposing that GS produces shorter chains that require subsequent elongation steps in the periplasmic space.

However, with improvements in purification methods (e.g. product entrapment using product affinity) and analytical techniques (especially SEC–PAD–RC capable of characterizing insoluble long-chain glucans), the estimation of product length was drastically revised. Analysis of Fks1 purified from a *S. cerevisiae* Δ*fks2* strain showed that the enzyme is capable of producing very long β-1,3-glucan chains, with an average DP as high as 6 550 ± 760, or in the range of 2000–7000. This length closely matches the DP values of β-1,3-glucan isolated from intact yeast cell walls. This strongly supports an alternative model, where GS directly synthesizes the full-length structural glucan backbone found in the cell wall. The product synthesized by purified GS *in vitro* is mostly, if not entirely, water-insoluble (Chhetri et al. 2020).

This revision regarding product length has significant implications for our understanding of cell wall biosynthesis models. Early models assumed GS produced short-chain precursors requiring extensive elongation by periplasmic enzymes (Shahinian and Bussey 2000). The new evidence indicates that GS directly synthesizes long-chain backbones close to their final length. This implies that the primary role of subsequent periplasmic enzymes (like Gas/Gel/Phr proteins might be more focused on branching, cross-linking, and integrating these long chains, rather than large-scale backbone elongation; Mouyna et al. 2000). This further underscores the central role of the Fks enzyme itself in determining the basic structure of the cell wall.

### TM transport of nascent glucan

The synthesized glucan polymer must be transported across the plasma membrane. The TM channel revealed by cryo-EM structures, located beneath the active site (see details in Glucan Translocation Channel), is considered the pathway for glucan extrusion (Hu et al. 2023).

The transport mechanism is thought to involve conformational changes in the enzyme, potentially coupled to substrate binding, catalysis, and Rho1 activation. These changes would open the channel, which is closed in the apo state, and facilitate the passage of the growing sugar chain. A specific model proposes that the movement of a “finger helix” triggered by Rho1 activation, which might push the glucan chain into the channel (Li et al. 2025).

The catalytic cycle of Fks appears to be a highly integrated process. Substrate binding, glycosyl transfer at the nonreducing end, processive elongation, and TM translocation must be tightly coupled. The active site must remain bound to the nonreducing end of the growing chain while feeding it into the translocation channel and accepting new UDP-Glc substrate. Conformational changes within the enzyme complex, such as the domain movements and channel gating revealed by structural studies, provide a possible physical basis for this coupling. Understanding this complex coupling mechanism is crucial for fully grasping the enzyme’s function and how inhibitors interfere with this process.

Fks enzyme uses UDP-Glc as a substrate to processively elongate the β-1,3-glucan chain at its nonreducing end. Recent studies indicate that Fks can directly synthesize very long glucan chains (DP up to thousands), close to the final length found in the cell wall, and translocate the nascent chain across the membrane via a dynamic channel within its TM domain. The entire catalytic and transport process is highly coupled, involving complex conformational changes of the enzyme.

## Inhibition of β-1,3-glucan synthase and antifungal resistance

### Fks as a premier antifungal target

The effectiveness of the Fks enzyme as an antifungal drug target has been validated by the clinical success of echinocandins and the development of ibrexafungerp (Nunnally et al. 2019). Inhibiting Fks activity leads to a weakened cell wall, osmotic imbalance, and fungal cell death.

### Echinocandins

This class includes cyclic lipopeptide compounds such as caspofungin, anidulafungin, micafungin, and so on (Denning, 2003). These compounds function as potent inhibitors of Fks enzymatic activity through a noncompetitive mechanism, thereby blocking β-(1→3)-glucan polymerization. Notably, even at high concentrations, echinocandins may not achieve complete inhibition of GS activity, with maximum inhibition rates plateauing around 70%–80% (Ko 2005, Debono and Gordee 2025). This indicates residual enzymatic functionality persists despite inhibitor binding and suggests inhibitors may not act directly on the Fks catalytic sites.

### Triterpenoids–ibrexafungerp

Ibrexafungerp is the first clinically approved drug of the “fungerp” class, a semisynthetic derivative of the natural product enfumafungin. It also exerts its antifungal effect by inhibiting β-1,3-glucan synthase (Schwebke et al. 2022, Goje et al. 2023). Ibrexafungerp is chemically distinct from echinocandins (Schwebke et al. 2022, Kumar et al. 2024). It is presumed to interact with the Fks enzyme differently, possibly binding to a site that overlaps but is not identical to the echinocandin site (Ghannoum et al. 2020). This might be the reason for its retained activity against some echinocandin-resistant strains.

### Structural basis of inhibition and resistance

Clinical resistance to echinocandins primarily arises from specific point mutations in the *FKS* gene. These resistance-associated mutations are not randomly distributed but cluster within three conserved “Hot Spot” regions (HS1, HS2, and HS3) (Johnson et al. 2011, Zajac et al. 2025). These regions may play critical functional or structural roles in drug binding, so mutations can reduce the inhibitory effect of echinocandins while allowing the enzyme to retain partial activity. However, structural studies clarifying the precise interactions between specific Fks1 hot spot regions and echinocandins remain lacking. Additionally, the structural basis for reduced caspofungin binding affinity caused by mutations at key residues, such as S643, remains unresolved.

**Figure 3.**
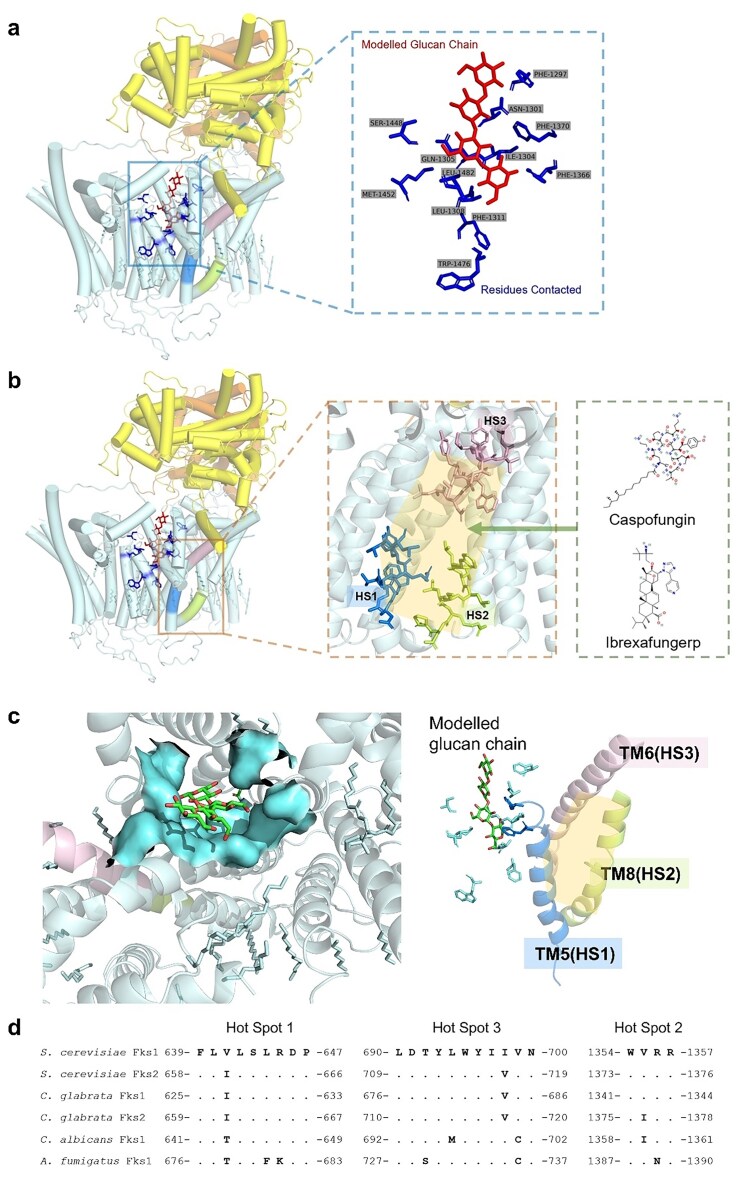
Architecture and relative positioning of the β-1,3-glucan translocation pathway and hot spots (putative drug-binding cavity) in *S. cerevisiae* Fks1. (a) The glucan translocation channel (cyan) in Fks1 (PDB: 7YUY), with key residues (blue sticks) lining the hydrophobic groove (red glucan chain). (b) A putative drug-binding cavity (orange) is framed by HS1, HS2, and HS3 (green, blue, and pink). The right panel shows structures of the echinocandin capsofungin and the tetrazole ibrexafungerp. (c) Glucan tunnel (cyan) directly abuts the drug pocket (HS1–HS3 helices, color-coded) (PDB: 7YUY). (d) The amino acid alignment of HS1–3 of *S. cerevisiae* Fks1/2, *C. glabrata* Fks1/2, *C. albicans* Fks1, and *A. fumigatus* Fks1.

Cryo-EM analysis shows that the glucan translocation channel formed by TM7, TM11, and TM12 abuts the drug-binding pocket, creating a functionally coupled architecture (Fig. 3a). In wild-type Fks1, this channel predominantly resides in a semiclosed conformation (Hu et al. 2023). Based on current structural and functional data, we annotated the three resistance hot spot regions (blue, green, and pink) in Fig. 3. These regions cluster together and likely represent putative drug-binding sites, although this hypothesis requires further validation through future structural biology studies. Spatial mapping demonstrates close proximity between the glucan transport channel and putative drug-binding site (HS1–HS3), separated only by TM8. Based on the observed structural features, we propose a model wherein drug binding to the pocket restricts opening of the adjacent glucan channel through putative allosteric coupling, thereby conferring antifungal activity. thereby indirectly constraining dynamic opening of the glucan channel. Such inhibition disrupts elongation and secretion of β-1,3-glucan chains, ultimately compromising fungal cell wall (CW) integrity. In contrast, drug-resistant mutants (e.g. S643P) exhibit lipid rearrangement near the channel that alters local flexibility. This structural perturbation likely attenuates the drug-induced conformational “locking” of the channel, consequently restoring glucan synthase activity. This proposed mechanism, however, requires future validation through structural biology studies.

Structural analysis of the echinocandin-resistant mutant fks1-S643P (S643 is in HS1) revealed altered arrangements of bound lipids near the mutation site (Hu et al. 2023). These structural insights suggest the following potential mechanisms for resistance: (1) direct alteration of drug binding: although the precise binding mode is still unclear (presumed to involve interaction of the drug’s lipid tail with both the Fks protein and membrane lipids), mutations might directly interfere with drug-target binding; (2) indirect effects: mutations might indirectly reduce inhibitor efficacy by altering the conformation, dynamics, or membrane interactions of the Fks protein.

The spatial overlap between resistance hotspots and the glucan translocation channel (formed by TM7–TM11–TM12) represents a structurally significant feature. It supports a dual mechanism of drug action: drug may not only by binding near the catalytic site but also disrupt conformational changes essential for glucan translocation or membrane-coupled enzyme dynamics. Resistance mutations in this critical region might subtly alter the enzyme’s dynamics or its interactions with the drug/membrane, allowing glucan extrusion to proceed even in the presence of the inhibitor.

**Figure 4.**
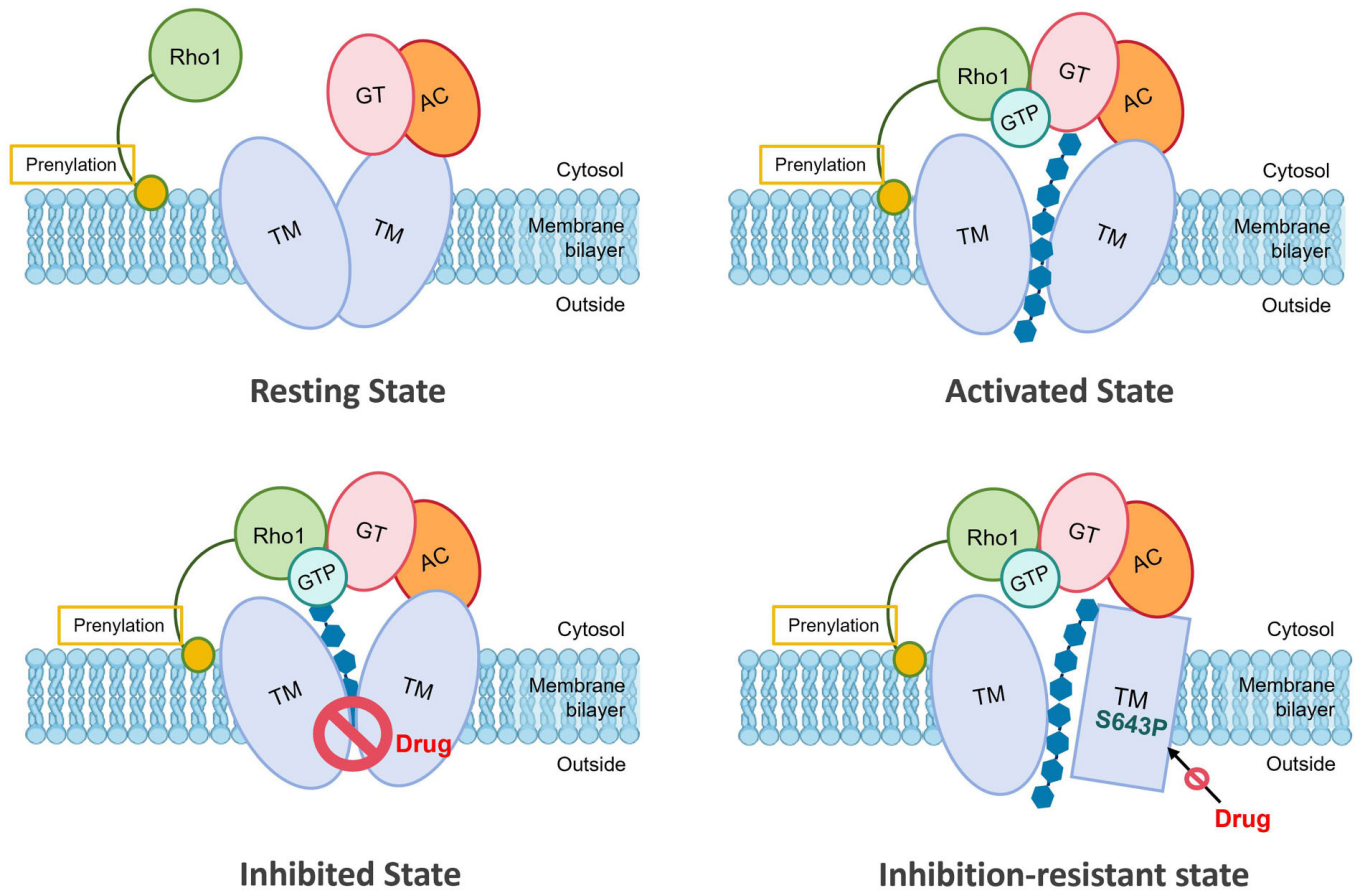
Conformational cycle of *S. cerevisiae* β-1,3-glucan synthase under Rho1 regulation with and without drug of echinocandins and triterpenoids. Four functional states: Resting: Rho1 unbounds to Fks1. Activated: GTP-bound Rho1 triggers glucan synthesis. Inhibited: Drug blocks glucan extrusion. Resistant: S643P mutation disrupts drug binding, enabling persistent synthesis.

Based on existing structural data and our analyses, we propose a functional and drug-resistance model for GS (Fig. 4). In this model, Fks1 does not act merely as a static channel but functions as part of a molecular “gearbox.” The regulatory subunit Rho1 serves as the on/off switch of GS, driving glucan chain elongation and active transport by binding to and inducing conformational changes in Fks1 (activated state). Echinocandin-class drugs bind to the TM helices of Fks1, interfering with the initiation of the glucan chain translocation channel. This disruption prevents the β-(1,3)-glucan chains synthesized by the GT domain from being extruded across the plasma membrane (inhibited state). Mutations in the three hotspot regions of Fks1 TM helices may reduce drug binding efficacy, thereby hindering drug-induced closure of the translocation channel. Alternatively, these mutations could alter the flexibility of the helices constituting the channel, enabling sustained opening of the glucan translocation pathway even in the presence of inhibitors (inhibition-resistant state).

### Other inhibitors

Besides clinically used drugs, other types of Fks inhibitors have been identified. For example, certain yeast-produced HM-1 killer toxins can inhibit β-1,3-glucan synthase activity extracellularly (Kasahara et al. 1994, Selvakumar et al. 2006). Furthermore, antiidiotypic scFv antibody fragments generated based on anti-HM-1 antibodies have also been shown to inhibit GS activity and fungal growth (Selvakumar et al. 2006).

The unique properties of ibrexafungerp—different chemical structure, distinct interaction mode with Fks, and effectiveness against some echinocandin-resistant strains—indicate the presence of multiple targetable binding sites or interaction modes on the Fks enzyme. If the binding sites and mechanisms were identical, higher cross-resistance would be expected. The proposed overlapping but nonidentical binding site (Ghannoum et al. 2020) provides a structural explanation: the two drugs might share some contact points but rely on different interactions for binding affinity or inhibitory effect. Mutations affecting echinocandin binding might not equally impact ibrexafungerp binding. This suggests that by targeting different regions of the enzyme or exploiting different conformational states, it might be possible to develop novel inhibitors capable of overcoming existing resistance mechanisms.

In summary, Fks is the key target for echinocandin and triterpenoid (ibrexafungerp) antifungal drugs. Resistance primarily arises from mutations in hotspot regions (HS1–3) of the *FKS* gene. Structural and molecular studies suggest echinocandins inhibit Fks by binding to a specific pocket in the TM region (near Hotspot and the glucan channel), while resistance mutations (like S643P) may weaken inhibition by altering local conformation, lipid interactions, or drug binding affinity. Ibrexafungerp likely interacts differently with Fks, providing a basis for its activity against some echinocandin-resistant strains and the possibility to develop novel inhibitors.

## Comparison of different GT protein function and structures

**Figure 5.**
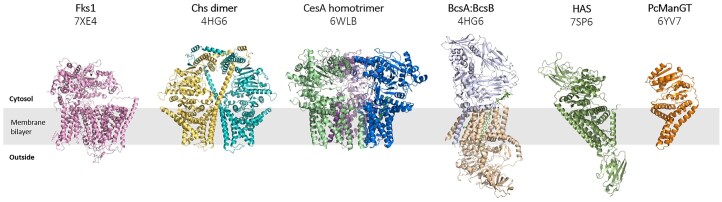
Comparison of different GT protein structures.

In this section, we provide a comparative analysis of fungal β-(1,3)-glucan synthase (Fks1), bacterial cellulose synthase (BcsA), plant cellulose synthase (CesA), chitin synthase (Chs), hyaluronan synthase (HAS), and mannosyltransferase (ManGT) based on published results (Fig. 5, Table 2).

**Table 2.** Comparison of representative membrane glycosyltransferases in polysaccharide synthesis.

Feature	Fks1 (β-1,3-glucan synthase)	Chs (chitin synthase)	CesA (plant cellulose synthase)	BcsA (bacterial cellulose synthase)	HAS (hyaluronan synthase)	ManGT (mannosyltransferase)
Representative species	*S. cerevisiae*	*S. cerevisiae, P. sojae, Aspergillus* spp.	*Populus tremula × P. tremuloides, Gossypium hirsutum*	*Cereibacter sphaeroides, Escherichia coli*	*Paramecium bursaria Chlorella virus (Cv-HAS)*	*Pyrobaculum calidifontis*
Family	GT48	GT2	GT2	GT2	GT2	GT2
Oligomeric state	Fks1–Rho1 hetero-dimer; Apo Fks1 monomer	Dimer	*In vitro*: monomer/trimer; *in vivo*: presumed hexameric rosette (composed of trimeric subunits)	BcsA–BcsB complex; larger multisubunit complex in *E. coli*; PDB 5EIY possibly monomer or dimer	Monomer (apo and substrate-bound states)	Monomer (crystallized as a monomer in apo and substrate-bound states)
Purification form	Recombinant purified (cryo-EM studies)	Recombinant-tagged protein (detergent micelle)	Recombinantly expressed in insect cells (purified with GDN etc.)	Recombinantly expressed in *E. coli* (structural studies)	Recombinant purified in *E. coli* lipid nanodiscs	Recombinant in E. coli (X-ray structure)
Key structural features	Cellulose synthase-like fold; GT-A domain; 15 TMs; TM channel; extracellular disulfide bonds	∼6 + TMHs; GT-A fold; transmembrane channel NTD (regulation/dimerization); MMD (Class V/VII, localization)	GT-A domain; 7–8 TMs; TM channel; Plant-specific regions (N-term zinc finger, P-CR, CSR)	8 TMs; cytoplasmic GT domain; PilZ domain; finger helix; gating loop; TM channel	GT-A domain; ∼6 TMHs, 3 Ifs; Substrate-binding pocket Transmembrane channel; Priming/switch loop Mn²⁺dependent catalysis	GT2 fold; divalent metal site; GT core domain
Regulatory mechanism	Rho1 GTPase activation; Drug inhibition	Zymogen activation; self-priming; localization; PTMs; dimerization	CSC assembly; Microtubule guidance; KOR1 interaction?; Hormone/environmental signals; UDP inhibition	c-di-GMP allosteric activation; ratcheting mechanism (finger helix/gating loop)	UDP-GlcNAc hydrolysis, GT domain changes, TM helices, substrate binding, channel opening,	Mn²⁺-dependent catalysis Dolichylphosphate (Dol-P) hydrolyzes GDP-α-Man in the absence of acceptor
Product	β-1,3-glucan (cell wall network component)	Microfibrils; fungal cell wall skeleton (cross-linked with β-glucan); insect exoskeleton	Cellulose (β-1,4-glucan); forms highly ordered microfibrils	Cellulose (β-1,4-glucan); can be modified (pEtN); forms fibrils/biofilms	Hyaluronan (HA; alternating β-1,3- and β-1,4-linked GlcNAc-GlcA heteropolysaccharide)	Transfers mannose from GDP-α-Man to dolichyldiphosphate (Dol-PP)-linked intermediates
PDB ID	7XE4, 7YUY, 8JZN 8WLA, 8WL6	7WJM, 8K52	6WLB, 8G27	5EIY, 4P02, 4HG6	7SP6, 7SP7, 7SP8, 7SP9 7SPA	6YV7
References	Li et al. (2025), Hu et al. (2023), Zhao et al.(2023)	Chen et al. (2022), Dorfmueller et al. (2014), Niu et al. (2024)	Verma et al. (2023), Kumar et al. (2017), Purushotham et al. (2020)	Morgan et al. (2014), Morgan et al. (2016), Acheson et al. (2021), Morgan et al. (2013)	Maloney et al. (2022)	Gandini et al. (2020)

The products of these enzymes are essential for their respective host organisms. For example, β-(1,3)-glucan and chitin form the structural basis of fungal CWs, maintaining their integrity (Lesage and Bussey 2006, Gow and Lenardon 2023); bacterial cellulose is a major component of biofilms and extracellular matrices, facilitating adhesion, colonization, and environmental adaptation (Morgan et al. 2014); plant cellulose serves as the primary load-bearing component of CWs, critical for cell morphogenesis, tissue development, and biomass formation (Cosgrove 2024). Additionally, hyaluronan (HA), synthesized by HAS, is an acidic heteropolysaccharide ubiquitous in the extracellular matrix of vertebrates (Górniak et al. 2025). Since fungal CW components are absent in mammalian cells, Fks1 and Chs have become vital targets for antifungal drugs, such as echinocandins and polyoxins. These polysaccharide synthases all belong to the GT superfamily.

According to the CAZy database classification system based on sequence similarity, BcsA, CesA, and Chs are typically classified under GT family 2 (GT2). GT2 family enzymes generally catalyze glycosidic bond formation via an inverting mechanism. Intriguingly, although Fks1 is classified under the GT48 family, structural studies reveal a cellulose synthase-like fold with a conserved GT-A-type GT domain (Hu et al. 2023). This structural similarity suggests that, despite differences in sequence-based classification, Fks1 may share conserved core catalytic and transport mechanisms with GT2 family members. However, structural comparisons reveal functional and regulatory divergences masked by this homology.

For instance, Fks1 synthesizes β-1,3-glucan, whereas BcsA and CesA produce β-1,4-glucan (cellulose). This difference in glycosidic linkage specificity necessitates distinct substrate orientation and bond formation mechanisms within their active sites. Furthermore, Fks1 employs a unique DAN motif for catalysis and operates independently of metal ions, contrasting with the canonical DxD motif and metal-dependent mechanisms of typical GT-A enzymes like BcsA. Additionally, Fks1 exhibits a specialized channel gating mechanism (e.g. inward shifts of TM8 in its apo state) and is regulated by the Rho1 GTPase—a fungus-specific adaptation absent in bacterial homologs. These differences highlight how Fks1 has diverged evolutionarily to meet the demands of fungal β-1,3-glucan synthesis and CW assembly, despite sharing a conserved structural framework with cellulose synthases.

Furthermore, HAS, a specialized GT, synthesizes HA, which plays diverse roles in extracellular matrices, including cell proliferation, migration, and tissue repair (DeAngelis and Zimmer 2023, Górniak et al. 2025). HAS polymerizes two distinct monosaccharides (*N*-acetylglucosamine and glucuronic acid) in an alternating manner to form HA. Like the other enzymes mentioned, HAS belongs to the GT superfamily, though its structure and mechanism may differ significantly from other family members.

Mannosyltransferase (ManGT) transfers mannose groups to acceptor molecules, participating in glycoprotein and glycolipid synthesis. These enzymes are crucial for cellular recognition, signaling, and intercellular interactions. While the precise structure and mechanism of ManGT remain incompletely characterized, its functional properties as a GT underscore its biological importance.

The structural and functional diversity of these enzymes reflects their evolutionary adaptations across different organisms. From a common ancestral enzyme, they have evolved highly specialized functions through domain acquisition, loss, or modification, as well as changes in oligomeric states, to meet the unique demands of their respective hosts. These enzymes are not only key to understanding fundamental biological processes such as CW/extracellular matrix biosynthesis and morphogenesis but also hold significant applied value. For instance, Fks1 and Chs, as antifungal drug targets, offer avenues for novel therapeutic strategies; bacterial and plant cellulose, as abundant renewable resources, inspire biotechnological applications (e.g. biomaterials and biofuels). HA and HAS also find extensive use in medicine and biomaterials. Continued exploration of these enzymes’ structures, functions, and regulatory mechanisms will yield further insights and opportunities for both basic biology and applied sciences.

Fks1 shares the core “cellulose synthase-like” fold with bacterial and plant cellulose synthases (GT-2 family) but exhibits significant divergence in product specificity (β-1,3 versus β-1,4 glycosidic bonds), catalytic mechanism (DAN motif versus DxD motif; metal ion independence), and regulation (Rho1 GTPase dependence). These adaptations reflect its evolutionary specialization for fungal β-1,3-glucan synthesis and CW construction, underscoring how structural homology can coexist with functional innovation.

## Regulation of β-1,3-glucan synthase activity, expression, and localization

The synthesis of β-1,3-glucan is subject to multilayered, precise regulation to ensure the CW is constructed and remodeled at the correct time and place.

### Activation by Rho1 GTPase

The activity of the Fks catalytic subunit is directly regulated by the Rho1 GTPase, one of the most important and conserved regulatory mechanisms. Enzyme activity strictly depends on Rho1 being in its GTP-bound activated state; GDP-bound Rho1 is inactive. Rho1’s own activity state is regulated by guanine nucleotide exchange factors (GEFs, e.g. Rom1/2) and GTPase-activating proteins (Smits et al. 2001).

Structural studies show that Rho1 bound to a GTP analog (GTPγS) binds in a pocket between the GT domain and the TM7–15 helix cluster of Fks1, and this binding is enhanced in the presence of GTPγS (Hu et al. 2023, Li et al. 2025). Rho1 binding induces extensive conformational changes in Fks1, particularly in the GT domain and TM7–17 region. An activation model has been proposed suggesting that Rho1’s GTP/GDP cycle acts like a “molecular pump” or “ratchet and pawl” mechanism, driving Fks1 dynamically between resting and activated states. The movement of a conserved “finger helix” might push the nascent glucan chain into the TM channel, thereby promoting β-1,3-glucan elongation.

### Signaling pathways and CWI

Fks activity and expression are tightly linked to cellular signaling pathways that monitor and maintain CWI (Liu and Balasubramanian, 2001).

Protein Kinase C (PKC) pathway is a key conserved pathway in fungi. CW stress sensors (e.g. Wsc family, Mid2) perceive perturbations in the CW and activate Rho1. Activated Rho1, on one hand, directly activates Fks, and on the other hand, activates PKC. PKC sits upstream of a Mitogen-activated protein kinase (MAPK) cascade, phosphorylating MAPKKK (e.g. Bck1) and relaying the signal down through phosphorylation (Mkk1/2 MAPKK→Slt2/Mpk1 MAPK), ultimately activating transcription factors (e.g. Rlm1) to regulate the expression of a range of CW-related genes. PKC might also directly phosphorylate other CW synthases, like chitin synthase (Gow et al. 2017).

Calcineurin pathway is involved in stress response and fungal virulence (Healey et al. 2020). In *S. cerevisiae, FKS2* expression is regulated by calcineurin. In *C. glabrata*, deleting the SSD1 gene, potentially linked to calcineurin signaling, affects Fks1 function or localization and leads to increased Fks2 expression (Healey et al. 2020). In *S. cerevisiae* Fks1 mutants can be highly sensitive to calcineurin inhibitors (like FK506, cyclosporin A).

The HOG pathway (high osmolarity glycerol pathway) also plays a role in regulating CW biosynthesis. And RIM101 Pathway (pH sensing pathway) is involved in CW regulation (Gow et al. 2017).

When the CW is damaged (e.g. by antifungal drugs like echinocandins), these signaling pathways are activated, triggering compensatory responses, typically including increased chitin synthesis and altered linkages between CW proteins and polysaccharides, leading to significant changes in CW architecture (Gow et al. 2017).

### Posttranslational modifications

Protein phosphorylation is a ubiquitous regulatory mechanism in eukaryotes (including fungi), controlling diverse cellular processes, including CW synthesis. Early biochemical studies suggested ATP-dependent phosphorylation events might synergize with GTP to regulate GS activity (Ariño et al. 2019). The PKC pathway itself involves extensive phosphorylation cascades (Kumar et al. 2024). Although the materials do not explicitly detail phosphorylation of Fks protein itself as its primary switch mechanism, considering that key kinases (like PKC) and phosphatases (like calcineurin) are involved in pathways regulating Fks expression and CWI, the possibility of Fks or Rho1 being regulated by phosphorylation is very high. Further research is needed to identify specific phosphorylation sites on Fks or Rho1 and their functional impact.

The regulatory subunit Rho1 requires prenylation (specifically geranylgeranylation) for proper membrane localization and activation of GS (Smits et al. 2001).

### Transcriptional regulation

The expression levels of *FKS* genes are precisely regulated: *In S. cerevisiae, FKS1* predominates during vegetative growth, while *FKS2* expression is upregulated during stress (high temperature and low glucose) or sporulation, regulated by calcineurin and the CWI pathway. FKS3 participates in sporulation. In *C. albicans, FKS*1 transcription is regulated by the cell cycle. Deletion of *FKS2* or *FKS3* leads to increased *FKS1* expression. Heterozygous deletion of *FKS1* does not affect *FKS2/FKS3* expression (Suwunnakorn et al. 2018). In *C. posadasii, FKS1* expression levels are similar in mycelia and early endosporulating spherules but decrease during spherule maturation (Kellner et al. 2005). Transcription factors activated in response to CW stress, such as Rlm1, Crz1 (calcineurin-dependent), Swi4/Swi6 (SBF), Msn2/Msn4, Ste12, Tec1, and so on, regulate the expression of a range of target genes, very likely including *FKS* genes themselves or other CWI-related genes (Gow et al. 2017).

### Cellular localization and trafficking

As integral membrane proteins, Fks proteins must be correctly transported and localized to specific regions of the plasma membrane, namely sites of active CW synthesis and remodeling.

In budding yeast, Fks1 localizes to sites of polarized growth: the prebud site, the tip of small buds, and the bud neck during cell division. Its localization often coincides with cortical actin patches. The regulatory subunit Rho1 also localizes to active growth sites (Smits et al. 2001). In fission yeast (*Schizosaccharomyces pombe*), its homolog Bgs1 localizes to growing cell ends, the contractile ring and septum during cell division, mating projections, cell fusion zones, and specific regions during spore wall synthesis and germination. Its correct localization depends on the actin cytoskeleton and septation initiation network proteins (Cortés et al. 2002). In *C. albicans*, surface-exposed β-1,3-glucan (indicating recent synthesis/remodeling activity) is mainly found at bud scars and punctate foci on the lateral walls of yeast cells (Yang et al. 2015). In *C. glabrata*, disruption of SSD1 might alter the localization or transport of Fks1 to the plasma membrane (Healey et al. 2020).

### Protein interactions

Besides the primary regulatory partner Rho1 (see details in Regulatory Subunit Rho1), Fks proteins might interact with other proteins. Copurification of *S. cerevisiae* GS using product entrapment yielded Gas1 in addition to Fks1 and Rho1. Gas1 is a β-1,3-glucanosyltransferase involved in glucan chain elongation and branching (Aimanianda et al. 2017). This finding suggests a possible physical interaction or close colocalization between the glucan synthase and glucan modifying enzymes. Future studies should employ modern proteomics approaches, such as quantitative affinity purification–mass spectrometry, to systematically characterize the Fks interactome and uncover novel regulatory components or connections to cellular machinery (Chen and Chen 2021).

The regulation of β-1,3-glucan synthesis is an extremely complex system operating at multiple levels, from gene expression to protein localization to direct enzyme activation (Gow et al. 2017). Rho1 provides direct, localized activation coupled to growth sites (Qadota et al. 1996). Signaling pathways (PKC, and so on) integrate internal and external signals to modulate the overall CWI response (Dichtl et al. 2012). Transcriptional regulation allows for long-term adaptive changes (Mazur et al. 1995). Precise localization ensures glucan deposition where needed (Wagener et al. 2020). And Post-translational modifications (PTMs, like phosphorylation and prenylation) offer rapid, reversible means of control(Mazur and Baginsky, 1996). No single mechanism dominates; it is the integration of these diverse regulatory inputs that ensures fungi can precisely control the amount, timing, and location of CW construction, enabling adaptation to different environments, morphological transitions, and responses to stress or damage (Gow et al. 2017, Gow and Lenardon 2023).

The activity, expression, and localization of the Fks enzyme are tightly regulated at multiple levels. Rho1 GTPase directly activates Fks activity; signaling pathways like PKC and calcineurin integrate CW stress signals; *FKS* gene expression is transcriptionally controlled; Fks protein must be precisely localized to cell growth sites; PTMs also participate in regulation. These regulatory networks collectively ensure the dynamic balance and integrity of the CW.

## Conclusions

Recent cryo-EM structures of fungal β-1,3-glucan synthase reveal its conserved GT-A fold and unique fungal adaptations, such as β-1,3-glucan synthesis via a DAN motif and Rho1 GTPase activation. Despite structural parallels to bacterial/plant cellulose synthases, Fks diverges in metal-independent catalysis and regulatory mechanisms. Critical unresolved questions include chain initiation mechanisms, inhibitor-binding details (e.g. echinocandins), species-specific structural diversity, and dynamic channel gating. Future priorities involve resolving Fks paralog structures, probing lipid interactions, and leveraging structural insights to design inhibitors targeting novel sites (e.g. Fks–Rho1 interface). Integrating static snapshots with *in vivo* dynamics and signaling pathways (PKC, calcineurin) will require advanced imaging techniques. These advances establish a foundation for understanding Fks function, resistance mechanisms, and therapeutic targeting.
